# Inhibition of MDM2 by Nilotinib Contributes to Cytotoxicity in Both Philadelphia-Positive and Negative Acute Lymphoblastic Leukemia

**DOI:** 10.1371/journal.pone.0100960

**Published:** 2014-06-26

**Authors:** Hailong Zhang, Lubing Gu, Tao Liu, Kuang-Yueh Chiang, Muxiang Zhou

**Affiliations:** Department of Pediatrics and Aflac Cancer and Blood Disorders Center, Emory University School of Medicine, Atlanta, Georgia, United States of America; University of Illinois at Chicago, United States of America

## Abstract

Nilotinib is a selective BCR-ABL tyrosine kinase inhibitor related to imatinib that is more potent than imatinib. Nilotinib is widely used to treat chronic myelogenous leukemia (CML) and Philadelphia-positive (Ph+) acute lymphoblastic leukemia (ALL). The present study identifies Mouse double minute 2 homolog (MDM2) as a target of nilotinib. In studying ALL cell lines, we found that the expression of MDM2 in both Philadelphia positive (Ph+) and Philadelphia negative (Ph-) ALL cells was remarkably inhibited by nilotinib, in a dose- and time-dependent manner. Further studies demonstrated that nilotinib inhibited MDM2 at the post-translational level by inducing MDM2 self-ubiquitination and degradation. Nilotinib-mediated MDM2 downregulation did not result in accumulation and activation of p53. Inhibition of MDM2 in nilotinib-treated ALL cells led to downregulation of the anti-apoptotic protein X-linked inhibitor of apoptosis protein (XIAP), a translational target of MDM2, resulting in activation of caspases. Inhibition of XIAP following nilotinib-mediated downregulation of MDM2 resulted in apoptosis of MDM2-expressing ALL; however, similar nilotinib treatment induced stronger apoptosis in Ph+/MDM2+ ALL than in Ph-/MDM2+ or Ph+/MDM2- ALL. The ALL cells that were Ph-/MDM2- were totally resistant to nilotinib. These results suggested that nilotinib can inhibit MDM2 and induce a p53-independent apoptosis pathway by downregulating XIAP; thus, nilotinib can treat not only Ph+, but also Ph- ALL patients whose cancer cells overexpress MDM2.

## Introduction

The tyrosine kinase inhibitors (TKIs) such as imatinib, dasatinib and nilitinib were designed and developed for the treatment of chronic myelogenous leukemia (CML) and certain acute lymphoblastic leukemia (ALL), based on the knowledge that the protein kinase ABL is constitutively activated in patients with these disease. It is known that the constitutive activation of ABL observed in CML and ALL patients is due to a t(9;21) chromosome translocation (Ph), in which the BCR protein becomes fused to ABL, to generate the BCR-ABL oncoprotein that drives both transformation and leukemogenesis [1]. This Philadelphia or Ph chromosome is found in almost all patients with CML and in 25-30% patients with ALL, where it produces a 210-KD and a 190-KD form of the BCR-ABL protein, respectively, in CML and ALL [2], [3]. BCR-ABL then activates a large number of signaling pathways in the cells [4], [5]; however, many of these pathways are thought to act in a redundant fashion, as only a few signaling components such as PI3K/AKT, NF-κB and STAT are reported to be critical for BCR-ABL-mediated oncogenic transformation and leukemogenesis [5]–[7].

Inhibition of the BCR-ABL tyrosine kinase activity by imatinib, the first generation of specific BCR-ABL inhibitors, results in durable cytogenetic and molecular remissions in the majority of CML and Ph+ ALL; however, not all patients benefit from treatment, due to drug resistance and intolerance [8], [9]. This led to the development of second-generation TKIs, such as nilotinib, which is more potent than imatinib and is currently approved for the treatment of newly diagnosed, imatinib-resistant or imatinib-intolerant CML and Ph+ ALL [10]. Although very successful hematologic and cytogenetic responses are obtained in nilotinib-treated patients, in recent years there have been cases with resistance to nilotinib [11], [12]. On the other hand, imatinib and nilotinib are also reported to induce apoptosis in BCR-ABL– cells of lymphoid origin [13] and are used for treatment of other types of cancer, such as advanced gastrointestinal stromal tumors [14]. The fact that some BCR-ABL+ cases are not sensitive to imatinib nor nilotinib and that some BCR-ABL– cancer cells are still killed by imatinib or nilotinib suggests the existence of additional pathways regulated by BCR-ABL in leukemia cell growth, and multiple mechanisms of action of imatinib and nilotinib in inducing cancer cell apoptosis. For example, it is reported that several cell survival and anti-apoptotic proteins such as BCL-X, survivin and histone deacetylases are downregulated by imatinib and nilotinib, which contribute to their cytotoxic and apoptotic activities [15]–[17].

MDM2, an oncoprotein, is also a regulatory target of BCR-ABL. BCR-ABL activates MDM2 mRNA translation [18] and the existence of MDM2 is required for the survival effects of BCR-ABL in hematopoietic cells [19]. In addition, c-ABL phosphorylates MDM2, facilitating formation of the MDM2-MDM4 complex, and MDM2 becomes stabilized in the MDM2-MDM4 complexes [20], [21]. The main oncogenic function of MDM2 is to inhibit the tumor suppressor p53 [22], [23]; thus, p53 function becomes inactivated in MDM2-overexpressing cells, resulting in cancer cell growth. MDM2 also plays p53-independent roles in oncogenesis. Increasing evidence suggests that even in p53-deficient cancer patients, MDM2 overexpression is still involved in cancer promotion and progression, as well as resistance to treatment [24]. This is because, in addition to interacting with and regulating p53, MDM2 interacts with other molecules involved in oncogenesis. We recently reported that binding of the C-terminal RING domain of the MDM2 protein to XIAP mRNA regulates translation of this apoptosis regulator, which allows for development of resistance to anticancer treatment [25]. Overexpression of MDM2 due to genomic amplification occurs in a variety of human cancers [26]. High levels of MDM2 expression can still be observed even in malignancies without MDM2 gene amplification, as occurs in leukemia [27]. Although no detailed studies on MDM2 expression in CML have been reported, it is known that overexpression of MDM2 is observed in 20 to 30% of patients with ALL [28]-[30] and is found to be associated with early treatment failure and a poor prognosis [31]–[34].

Because MDM2 is positively regulated by c-ABL and BCR-ABL, we evaluated whether inhibition of the ABL activity by nilotinib had effects on MDM2 expression. We found that nilotinib strongly inhibited MDM2 expression and induced apoptosis of both BCR-ABL positive and negative ALL cells. Herein, we delineate the mechanism by which nilotinib inhibits MDM2 expression and the mechanistic steps involved in MDM2 downregulation that induce ALL cell apoptosis.

## Materials and Methods

### Cells and reagents

This study used six established cell lines derived from children with ALL. Four of these cell lines (EU-1, EU-5, EU-6 and EU-9) were established at Emory University by Dr. Muxiang Zhou, and two (UOC-B1 and SUP-BI5) were established at University of Chicago by Dr. Stephen D. Smith. All cell lines were authenticated and their phenotypes, including the status of p53 and BCR-ABL as well as MDM2 expression levels, were characterized by Drs. Zhou and Smith in prior publications [28], [35]. All cell lines grew in standard culture medium (RPMI 1640 containing 10% FBS, 2 mM L-glutamine, 50 U penicillin and 50 µg/ml streptomycin), at 37°C in 5% CO_2_. The nilotinib (>98% pure) used was purchased from Caymanchem (Ann Arbor, MI, USA), and stock solution was prepared at a concentration of 10 mM, in deionized water. The treatment of cells was by exposure to 0.25–5 µM of nilotinib, for the time periods given. Antibody to Bcr-Abl (Ab-3) was purchased from Calbiochem. We also purchased antibody to MDM2 (SMP14) from Sigma, antibody to p53 (DO-1) from Santa Cruz, antibodies to p21 (12D1) and PARP (7D3-6) from Cell Signaling, and antibody to XIAP (2F1) from Abcam. The concentrations of all antibodies were used according to the manufacturers' instructions. The caspase-3 and caspase-9 ELISA kits were purchased from Enzo.

### Plasmid, siRNA and transfection

The XIAP expression plasmid pCDNA3-6myc-XIAP was provided by Dr. L. Yang (Emory University). For XIAP gene transfection, SUP-B15 cells in exponential growth were transfected with pCDNA3-6myc-XIAP plasmid and vehicle control plasmid by electroporation at 300 V, 950 µF using a Gene Pulser II System (Bio-Rad, Hercules, CA). The p53 siRNA and control siRNA were purchased from Santa Cruz. Transfection of these siRNAs was performed using the HiPerFect transfection reagent (Qiagen), following the manufacturer's manual.

### Immunoprecipitation (IP) and western blot assay

Cells were lysed in a buffer composed of 50 mM Tris-HCl, pH 7.6, 150 mM NaCl, 1% Nonidet P-40, 10 mM sodium phosphate, 10 mM NaF, 1 mM sodium orthovanadate, 2 mM phenylmethylsulfonyl fluoride (PMSF), 10 µg/ml aprotinin, 10 µg/ml leupeptin and 10 µg/ml pepstatin. After centrifugation, the clarified cell lysate was incubated with 15 µl Protein G plus/Protein A-agarose and 1 µg of antibodies, overnight at 4°C. Cell lysates or immunoprecipitates were resolved by sodium dodecyl sulfate - polyacrylamide gel electrophoresis (SDS-PAGE). Following transfer of the gel contents to a nitrocellulose filter, the filter was blocked for 1 h at room temperature, with buffer containing 20 mM Tris-HCl (pH 7.5), 500 mM NaCl and 5% non-fat milk; then incubated with specific antibodies for 3 h at room temperature, washed, and incubated with a HRP-labeled secondary antibody for 1 h. Finally, the blots were developed using a chemiluminescent detection system (ECL, Amersham Life Science, Buckinghamshire, England).

### Reverse transcription (RT)-PCR

The total RNA was extracted with the RNeasy Mini Kit (Qiagen, Hilden, Germany). For testing MDM2 mRNA expression, First-strand cDNA synthesis was performed with mixture of random nonamers and oligo-dT as primers (Qiagen). Amplification of MDM2 was performed with a 7500 Real-Time RT-PCR System (Applied Biosystems, Foster City, CA), using QuantiFast SYBR Green RT-PCR kit (Qiagen) according to the manufacturer's instructions. The MDM2 primers and the house-keeper gene GAPDH and Actin were purchased from Qiagen (sequences of the primers not provided).

### Pulse-chase assay

The protein turnover was assessed by a standard protein-synthesis inhibitor cycloheximide (CHX) assay. Briefly, cells were treated with 50 µg/ml CHX for different times before lysis, in the presence or absence of nilotinib. Western blot analysis of the products revealed concurrent expression levels of MDM2.

### Polysome preparation and analysis

Polysome profiling was carried out essentially as described previously [25]. Briefly, SUP-B13 cells, with or without exposure to nilotinib, were incubated with 100 µg/ml CHX for 15 min to arrest polyribosome migration. Cells were then lysed to isolate cytoplasmic RNA in a buffer containing 20 mM Tris-HCl at pH 8.0, 100 mM NaCl, 5 mM MgCl_2_, 0.5% Triton X-100, 500 U/ml RNAsin, and a cocktail of protease inhibitors, followed by fractionation on 15–45% (w/v) sucrose gradient. The gradient was centrifuged in a SW41Ti rotor at 39,000 rpm for 1 hr. Fractions were collected from each gradient tube by up-ward replacement with monitored absorption at OD_254_ by using a fractionator (Brandel, Inc). The RNA in each fraction was extracted and subjected to quantitative PCR as described above.

### WST assay

The cytotoxic effect of triptolide on ALL cells was determined using the water-soluble tetrazolium salt (WST) assay. Briefly, cells cultured in 96-well microtiter plates were given different concentrations of triptolide, for a 20-h period. Following this, WST (25 µg/well) was added and incubation continued for an additional 4 h before the optical density (OD) of the wells was read with a microplate reader (set at a test wavelength of 450 nm and a reference wavelength of 620 nm). Appropriate controls lacking cells were included, to determine background absorbance.

### Flow cytometry

Flow cytometry was performed to analyze the cell cycle position and degree of apoptosis induced by nilotinib. For the cell-cycle analysis, cells were collected, rinsed twice with phosphate buffered saline (PBS), fixed in 70% ethanol for 1 h at 4°C, washed twice in PBS and re-suspended in 0.5 ml PBS containing 20 µg/ml of propidium iodide (PI) and 20 µg/ml of RNase A. Following incubation at 4°C for at least 30 min, the samples were analyzed using a FACScan (Becton Dickinson) with WinList software (Verity Software House, Inc.).

For the quantitative detection of apoptotic cells, flow cytometry of annexin-V stained cells was performed. Briefly, cells with or without nilotinib treatment were washed once with PBS and stained with Annexin-V and PI, according to the manufacturer's instructions, prior to flow analysis.

### Statistical analysis

All comparative data was statistically analyzed using Statistical Package for the Social Sciences (SPSS) software. The differential significance was determined via Student's *t*-test and presented the data as the mean ± SD. The statistically significant difference was considered at *p*<0.05.

## Results

### Nilotinib inhibits MDM2 expression in ALL

**Figure 1:**
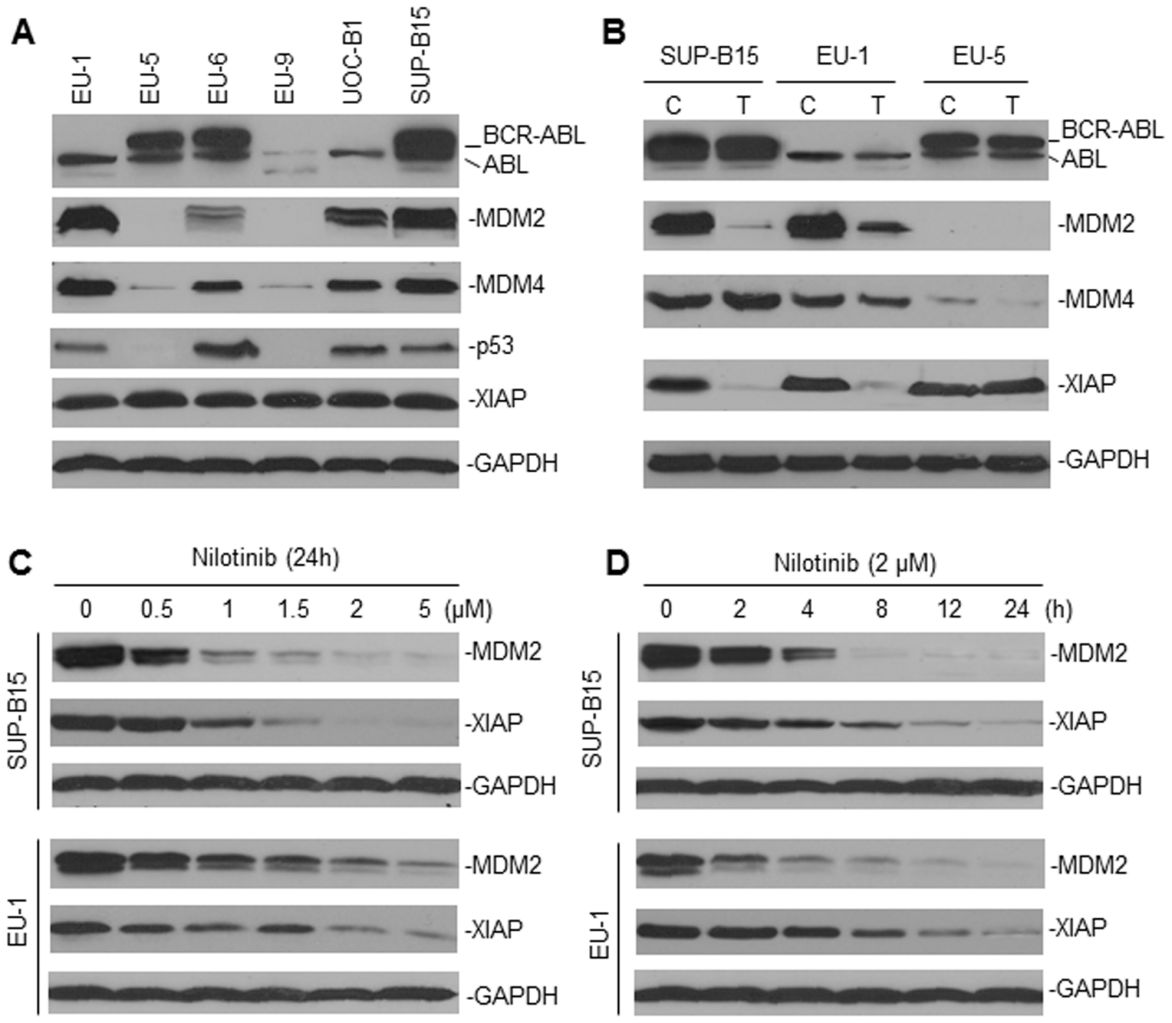
Downregulation of MDM2 by nilotinib in ALL. **A**, western blot assay for the expression of BCR-ABL, MDM2, p53 and XIAP in six cultured ALL cell lines. The p53 status of these lines (EU-1, UOC-B1 and Sup-B15: wt-p53; EU-6: mutant-p53; EU-5 and EU-9: p53-null) was previously characterized [28]. **B**, changes of the expression levels of MDM2 and XIAP by nilotinib treatment. Representative cell lines, as indicated, were treated (T) with 2 µM nilotinib for 24 h. Untreated cells served as a control (C). The expression of proteins was detected by western blot, as indicated. **C** and **D**, western blot assays showed the dose-response and time-course of MDM2 and XIAP inhibition by nilotinib, in both BCR-ABL positive (SUP-B15) and negative (EU-1) ALL cell lines.

To investigate whether nilotinib can inhibit MDM2, we tested a group of ALL cell lines (three were BCR-ABL+ and three were BCR-ABL-) that were known to express different levels of MDM2 (Fig. 1) and have various p53 status (EU-1, UOC-B1 and SUP-B13: wild-type (wt)-p53; EU-6: mutant p53; EU-5 and EU-9: p53-null) [28]. First, we found that there seemed to be no correlation between BCR-ABL expression and the MDM2 protein levels in ALL. As seen in Fig. 1A, overexpression of MDM2 and lack of MDM2 expression were detected in both BCR-ABL positive and negative ALL. Also, the BCR-ABL+ cell lines either had wt, mutant or had a null p53 phenotype, whereas high-level MDM2 expression was always associated with an enhanced expression of MDM4 and with a wt-p53 phenotype. All six cell lines studied expressed similar levels of XIAP. Treatment with nilotinib potently downregulated MDM2 expression in two MDM2-overexpressing ALL cell lines (Fig. 1B). The protein levels of either BCR-ABL or ABL appeared not to be changed by nilotinib. Also, MDM4 expression was not significantly affected by nilotinib treatment. Nilotinib inhibited the expression of XIAP in the two MDM2-overexpressing cell lines, but not in the MDM2-negative line, suggesting that inhibition of XIAP by nilotinib is MDM2-dependent. Nilotinib inhibited MDM2 and XIAP expression in a dose-dependent manner, spanning 0.5–5 µM (Fig. 1C). Inhibition of MDM2 and XIAP by nilotinib occurred at about 2–4 h after treatment and was followed by steady-state downregulation (Fig. 1D).

### Nilotinib downregulates MDM2 at the post-translational level

**Figure 2:**
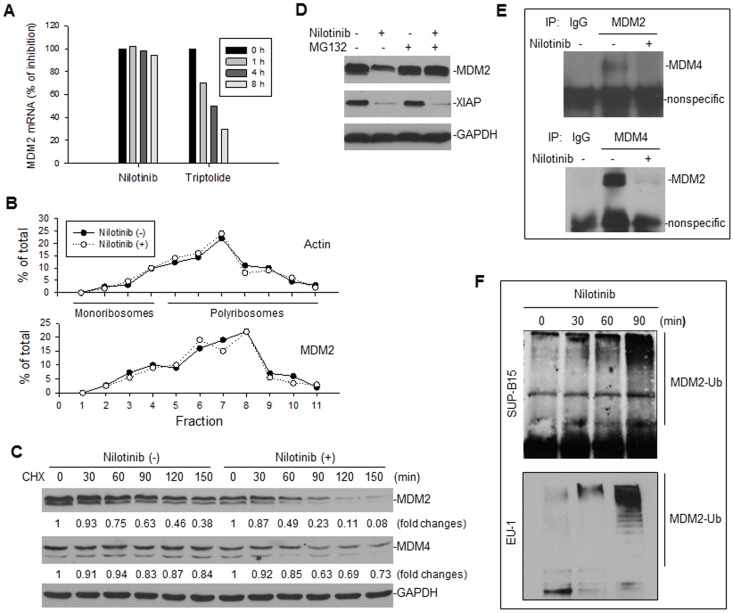
Evaluation of the mechanism by which nilotinib inhibits MDM2. **A**, RT-PCR for MDM2 mRNA expression in SUP-B15 cells, treated with 2 µM nilotinib and 0.2 µM triptolide (as a control) for the different times, as indicated. **B**, SUP-B15 cells were treated with or without 2 µM nilotinib for 4 h; then their cytoplasmic lysates were fractionated on a sucrose gradient. RNA was extracted from each of the fractions and subjected to quantitative RT-PCR for analysis of the distribution of MDM2 and actin mRNAs. Data represent the percentage of the total amount of corresponding mRNA in each fraction. **C**, CHX pulse-chase assay for detection of MDM2 turnover in SUP-B15 cells treated with or without 2 µM nilotinib for 4 h. Numerical labels under each protein band represent protein expression levels after normalization for GAPDH, compared with the untreated (0) samples (defined as 1 unit). **D**, SUP-B15 cells with or without nilotinib exposure were treated with 10 µM MG32 for 4 h and then the expression of proteins as indicated was assessed by Western blot. **E**, co-IP and western blot assay to detect the effect of nilotinib on the interaction between MDM2 and MDM4. Cell lysates from SUP-B15 treated with or without nilotinib were immunoprecipitated with the indicated antibodies. Normal mouse IgG served as a control. Proteins in immune complexes were separated on denaturing gels, transferred to filters, and then detected by western blotting with antibodies, as indicated. Antibodies for western blotting were from different species than those used in the IP. **F**, IP-western blot assay for detection of MDM2 ubiquitination after nilotinib treatment. SUP-B15 cells were treated with 2 µM nilotinib for the indicated length of time. Cellular lysates were immunoprecipitated with anti-MDM2 antibody, and then the MDM2 ubiquitination (MDM2-Ub) was detected by Western blot using anti-ubiquitin antibody.

We wished to investigate further how MDM2 is regulated by nilotinib. First, we performed quantitative RT-PCR for the expression of MDM2 mRNA in nilotinib-treated SUP-B15 cells. We found that nilotinib did not inhibit MDM2 mRNA expression (Fig. 2A), as compared with a previously identified MDM2 inhibitor triptolide, which downregulates MDM2 through inhibition of mRNA expression [36]. Nilotinib also did not inhibit MDM2 mRNA expression in the BCR-ABL– line, EU-1 (data not shown). Because a previous study demonstrates that BCR-ABL increases MDM2 mRNA translation [18], we investigated whether presumable inhibition of BCR-ABL by nilotinib in SUP-B15 cells could result in a decrease of MDM2 mRNA translation. In our experimental mode and setting, we did not detect an effect of nilotinib on MDM2 mRNA translation, because we detected no effect of nilotinib on the polyribosome profile of MDM2 (Fig. 2B). Furthermore, we measured the turnover of MDM2 after nilotinib treatment by pulse-chase assay. The half-life of MDM2 in untreated cells was larger than 90 min, whereas treatment with nilotinib decreased the MDM2 half-life to <60 min (Fig. 2C), indicating that nilotinib treatment induced MDM2 protein degradation. Nilotinib did not significantly alter the half-life of MDM4. The nilotinib-induced degradation of MDM2 was blocked by the proteasome inhibitor MG132 (Fig. 2D). As also seen in this figure, downregulation of XIAP was not inhibited by MG132, indicating that nilotinib-mediated inhibition of XIAP did not occur at the post-translational level.

Previous studies show that MDM2 protein degradation is regulated by an autolytic mechanism, through ubiquitination, and that MDM2 becomes unstable when it is dissociated from the MDM2-MDM4 complex [21]. Also, previous studies demonstrate that phosphorylation of MDM2 by c-BAL facilitates MDM2-MDM4 complex formation [20]. Thus, we tested whether nilotinib, which inhibits BCR-ABL and c-ABL, is able to block the MDM2-MDM4 interaction and induce MDM2 ubiquitination. As expected, co-IP/western blot assay results showed that the MDM2-MDM4 complexes were disrupted by nilotinib treatment (Fig. 2E), resulting in ubiquitination of MDM2 in both BCR-ABL positive and negative cells (Fig. 2F).

### The effects of nilotinib on p53 and XIAP functions

**Figure 3:**
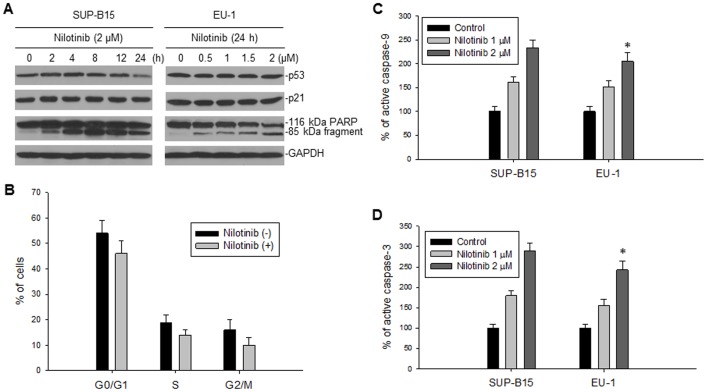
The effect of nilotinib-mediated downregulation of MDM2 and XIAP on activation of p53 and caspases. **A**, SUP-B15 (BCR-ABL+) and EU-1 (BCR-ABL–) were treated with 2 µM nilotinib for different times and different doses for 24 h, respectively. The expression of p53, p21 and PARP was detected by western blotting. **B**, cell-cycle analysis in SUP-B15 cells performed 4 h post-treatment with 2 µM nilotinib, as compared with untreated cells. **C** and **D**, activation of caspase-9 (C) and -3 (D) in nilotinib-treated SUP-B15 and EU-1 cells was detected by enzyme-linked immunosorbent assay (ELISA), *p>0.05.

Because MDM2 is an inhibitor of p53, we investigated the consequences of nilotinib-mediated downregulation of MDM2 on p53 expression. As is seen in Fig. 3A, the protein levels of p53 were not significantly changed by nilotinib in two wt-p53 cell lines with BCR-ABL (SUP-B15) or without BCR-ABL (EU-1) expression. Consistent with having no change in p53 expression, there was neither detectable alteration of p21 protein levels (Fig. 3A) nor G1 cell-cycle arrest (Fig. 3B) in these nilotinib-treated ALL cells. These results suggest that neither p53 accumulation nor activation occur following nilotinib-mediated-downregulation of MDM2. However, we detected a significant cleavage of the death substrate PARP in both cell lines after nilotinib treatment (Fig. 3A). Because PARP cleavage is always a result of activation of caspases such as caspase-9 and -3, we looked for activation of these caspases in nilotinib-treated cells: caspase-9 and -3 were indeed activated in both BCR-ABL positive and negative ALL cells (Fig. 3C and Fig. 3D), suggesting that the function of XIAP is inhibited by nilotinib, because XIAP specifically inhibits both these caspases [37], [38]. In comparing the activation levels of caspase-9 and -3 induced by nilotinib between BCR-ABL positive and negative ALL, we did not detect any significant differences, as *p*>0.5 (Fig. 3C and D).

### Nilotinib induced different degrees of apoptosis in ALL with distinct BCR-ABL and MDM2 expression

**Figure 4:**
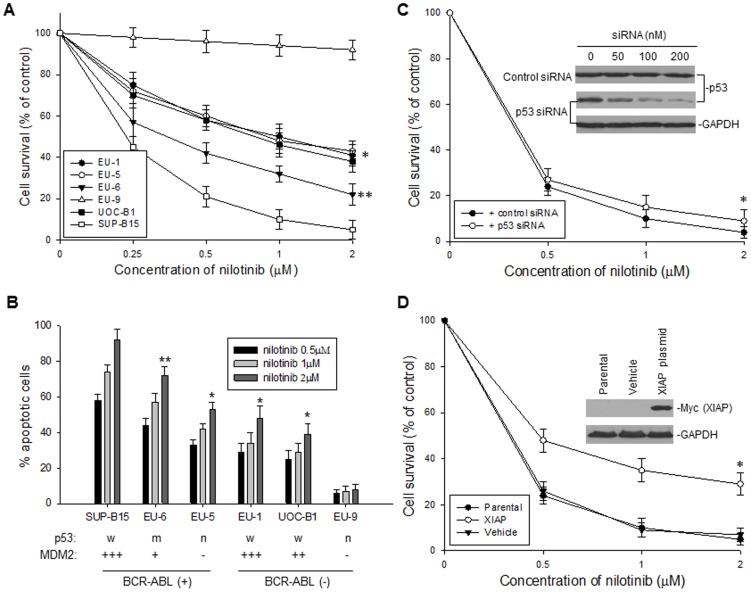
Cytotoxic and apoptotic effects of nilotinib on ALL cells. **A**, dose-dependent cytotoxic response to nilotinib in the six ALL cell lines, as tested for Fig. 1A. The viability of cells incubated with different concentrations of nilotinib for 24 h was determined by WST assay. Data from three independent experiments is represented by the mean percentage ± SD of surviving cells, as compared to the untreated controls. *p<0.01; **p<0.05 (as compared with SUP-B15) **B**, dose-response of apoptosis of the six cell lines treated with the nilotinib doses indicated for 24 h, as quatitatively detected by flow cytometry. *p<0.01; **p<0.05 (as compared with SUP-B15). **C**, the effect of p53 knockdown on sensitivity of BCR-ABL+ cells to nilotinib. SUP-B15 cells, transfected with 200 nM of either p53 siRNA or control siRNA, were treated with different dose of nilotinib for 24 h, and cell viability was detected by WST assay, *p>0.5. The expression of p53 in SUP-B15 cells transfected with p53 siRNA was determined by Western blot assay (*insert*). **D**, the effect of enforced overexpression of XIAP on sensitivity of ALL cells to nilotinib. SUP-B15 cells, transfected with pCDNA3-6myc-XIAP plasmid and vehicle control, were treated with different dose of nilotinib for 24 h, and cell viability was detected by WST assay, *p<0.05. Western blot showing the expression of ectopic XIAP (as detected by Myc antibody) in SUP-B15 cells (*insert*).

To investigate where nilotinib-mediated downregulation of MDM2 contributes to nilotinib's cytotoxic and apoptotic activities, we evaluated and compared the cytotoxic and apoptotic effects of nilotinib on ALL cells with different BCR-ABL and MDM2 expression status. We found that the degree of cytotoxicity and apoptosis induced by nilotinib in BCR-ABL+ cells was related to their expression levels of MDM2. WST cytotoxic assay results showed that nilotinib exhibited very strong cytotoxic effects on the BCR-ABL+ ALL cell line SUP-B15, which overexpresses MDM2 (Fig. 4A); while EU-6 cells with lower expression of MDM2 had relatively low sensitivity to nilotinib (*p*<0.05); and EU-5 cells with no expression of MDM2 had much lower sensitivity (*p*<0.01), when compared with SUP-B15. Even in the BCR-ABL– ALL cells, the cytotoxic effects of nilotinib were associated with the expression of MDM2, so the EU-9 BCR-ABL– ALL cells with no MDM2 expression were totally resistant to nilotinib. We also tested for induction of apoptosis by nilotinib in the 6 cell lines, by annexin-V and PI staining and flow cytometry. In consistency with the results of the WST assay, nilotinib induced very strong apoptosis in SUP-B15 (BCR-ABL+, MDM2 overexpressing) and less apoptosis in those cell lines that were either BCR-ABL+ with no and low levels of MDM2 expression, or BCR-ABL– cell lines with MDM2 overexpression. We detected no apoptotic effect for nilotinib-treated EU-9, a BCR-ABL– and MDM2– cell line (Fig. 4B).

We presented the data in Fig. 3A and 3B showing that neither p53 expression nor p53 function was altered by nilotinib, suggesting that the nilotinib-induced cytotoxicity and apoptosis are p53-independent. To confirm this, we performed an experiment using p53 siRNA to knock down p53 in SUP-B15 and treated these cells with nilotinib. Inhibition of p53 did not significantly alter the sensitivity of SUP-B15 to nilotinib (Fig. 4C). In contrast, our data in Fig. 1 and 3 apparently indicated a subsequent inhibition of XIAP expression and function following nilotinib-mediated downregulation of MDM2. We transfected a XIAP expression plasmid into SUP-B15 cells, and then treated the cells with nilotinib. Because the expression of the transfected XIAP was not driven by the XIAP Internal ribosome entry site (IRES), downregulation of MDM2 did not inhibit its expression. Cell death triggered by nilotinib was significantly attenuated by the enforced overexpression of ectopic XIAP (Fig. 4D), which suggests that inhibition of XIAP indeed confers, at least in part, to apoptosis induced by nilotinib.

## Discussion

Although the use of second-generation TKIs such as nilotinib to treat imatinib-resistant Ph+ ALL significantly improved the clinical outcome, there still existed cases that failed to respond to nilotinib. In a clinical trial of 126 patients treated with nilotinib after imatinib failure, the complete hematologic and major cytogenetic responses reported are only 33% and 50%, respectively, in Ph+ ALL [39]. This suggests that additional mechanisms exist in Ph+ ALL for resistance to nilotinib. For example, the *Ikaros* gene is identified as frequently deleted in Ph+ ALL, which is associated with resistance to TKIs [40], [41]. In the present study, we demonstrated that the expression level of MDM2 in ALL seems to be associated with the sensitivity of the cells to nilotinib. The ALL cell lines having both MDM2 overexpression and BCR-ABL expression were found to be very sensitive to nilotinib, whereas BCR-ABL-expressing ALL lines with no or low levels of MDM2 expression were less sensitive to nilotinib. We discovered that nilotinib specifically and strongly induced degradation of MDM2, through disruption of the interaction between MDM2 and MDM4; however, downregulation of MDM2 in nilotinib-treated cells did not result in activation of p53. Instead, we observed a reduction of XIAP expression and activation of caspase-3 and -9, following MDM2 inhibition in these nilotinib-treated cells, leading to their apoptosis.

The expression of MDM2 is regulated mainly at the post-translational level, through a self-ubiquitination mechanism, although previous studies report that MDM2 is transcriptionally activated by p53 [42] and translationally induced by BCR-ABL [18]. In the set of ALL cell lines that we studied, there was an association of MDM2 overexpression with wt-p53, while we detected no correlation with regard to the levels of MDM2 with BCR-ABL expression. Treatment of these cell lines with nilotinib detected neither reductions of p53 nor decreases in BCR-ABL and ABL expression. However, nilotinib remarkably inhibited MDM2 expression in all the MDM2-expressing cell lines, including the BCR-ABL positive and negative lines. We performed RT-PCR and polysome profiling assays, finding that nilotinib-mediated downregulation of MDM2 did not occur at the transcriptional nor translational levels. We observed that the half-life of the MDM2 protein was significantly reduced in nilotinib-treated cells. Further studies showed that the MDM2 and MDM4 complexes were separated by nilotinib; thus, increased ubiqitination of MDM2 was detected in these nilotinib-treated cells.

A well-characterized finding is that c-ABL phosphorylates MDM2, which facilitates the formation of the MDM2-MDM4 complex [20], where MDM2 becomes stabilized [21]. Our results indicating that nilotinib, the ABL inhibitor, disrupted the MDM2-MDM4 interaction and induced MDM2 degradation, seems a validation of the previous observation. It is easy to understand that the constitutive activation of ABL in BCR-ABL+ ALL is inhibited by nilotinib, which leads to the dissociation of MDM2 from the MDM2-MDM4 complex, resulting in ubiquitination and degradation; however, we also detected an inhibition of MDM2 by nilotinib in the BCR-ABL– ALL. This suggested that inhibition of the normal c-ABL activity by nilotinib in any BCR-ABL– cells was adequate to block the interaction between MDM2 and MDM4, resulting in MDM2 degradation. The degradation of MDM2 is most likely the mechanism for nilotinib to induce the apoptosis of BCR-ABL– cells.

The expression level of MDM2 seemed to play a partial role in induction of apoptosis by nilotinib. For example, the BCR-ABL– cell lines EU-1 and UOC-B1, which express high levels of MDM2, were sensitive to nilotinib; whereas the EU-9 cell line lacking both BCR-ABL and MDM2 expression was not killed by nilotinib. Most normal cells/tissues express no or very low levels of MDM2, which is probably the reason for nilotinib to be developed as a drug for clinical use.

Previous studies also demonstrate that c-ABL is a positive regulator of p53 [43], interacting with p53, which can activate its downstream target p21 [44]. Treatment of CML with imatinib impairs p53 accumulation and function [45]. Similarly, we did not detect an induction of p53 after downregulation of its inhibitor MDM2 in the nilotinib-treated cell lines, both BCR-ABL positive and negative. This could be explained by considering that the induction of p53 and its target p21, due to inhibition of MDM2, is diminished or abrogated by the direct inhibitory effect nilotinib has on p53 and p21, through its inhibition of BCR-ABL or c-ABL. Thus, neither p53 activation nor inhibition was detected in the nilotinib-treated cells.

MDM2 also regulates cancer cell growth and resistance to apoptosis in a p53-independent manner. For example, MDM2 is a positive regulator of the anti-apoptotic factor XIAP, binding the XIAP IRES to induce XIAP mRNA translation, which results in resistance of cancer cells to radio-chemotherapy [25]. Inhibition of MDM2 by specific siRNA or specific inhibitors, such as triptolide, is always followed by a downregulation of XIAP [25], [36]. In the present study, we also found a concomitant downregulation of XIAP and activation of caspase-9 and caspase-3, following nilotinib-mediated inhibition of MDM2. Based on our results, we now believe that the potent apoptosis induced by nilotinib in BCR-ABL+ and MDM2-overexpressing ALL cells are partially attributable to the downregulation of XIAP. In contrast, lack of XIAP downregulation in BCR-ABL+ cells due to loss of their MDM2 expression, which usually occurs in p53-deficient cancer cells such as the EU-5 cell line, could be one of the mechanisms for these cells' resistance to nilotinib.

The significance of the present study is our finding that nilotinib can inhibit MDM2 expression in BCR-ABL– ALL cells. Overexpression of MDM2 is detected in about 20-30% of ALL and is associated with refractory disease. Although nilotinib is unable to induce the activation of p53 in the MDM2-overexpressing ALL, it can induce the downregulation of the anti-apoptotic protein XIAP, following its inhibition of MDM2. Thus, we believe nilotinib should be an interesting candidate drug for therapy against high-risk, refractory ALL, including in Ph– patients whose leukemic cells overexpress MDM2.
